# Diet and body constitution in relation to subgroups of breast cancer defined by tumour grade, proliferation and key cell cycle regulators

**DOI:** 10.1186/bcr1644

**Published:** 2007-01-25

**Authors:** Signe Borgquist, Elisabet Wirfält, Karin Jirström, Lola Anagnostaki, Bo Gullberg, Göran Berglund, Jonas Manjer, Göran Landberg

**Affiliations:** 1 Division of Pathology, Department of Laboratory Medicine, Malmö University Hospital, S-205 02 Malmö, Sweden; 2 Division of Oncology, Department of Clinical Sciences, Lund University Hospital, S-221 85 Lund, Sweden; 3 Department of Clinical Sciences, Malmö University Hospital, S-205 02 Malmö, Sweden; 4 Department of Surgery, Malmö University Hospital, S-205 02 Malmö. Sweden

## Abstract

**Background:**

The general lack of clear associations between diet and breast cancer in epidemiological studies may partly be explained by the fact that breast cancer is a heterogeneous disease that may have disparate genetic associations and different aetiological bases.

**Method:**

A total of 346 incident breast cancers in a prospective cohort of 17,035 women enrolled in the Malmö Diet and Cancer study (Sweden) were subcategorized according to conventional pathology parameters, proliferation and expression of key cell cycle regulators. Subcategories were compared with prediagnostic diet and body measurements using analysis of variance.

**Results:**

A large hip circumference and high body mass index were associated with high grade tumours (*P *= 0.03 and 0.009, respectively), whereas low energy and unadjusted fat intakes were associated with high proliferation (*P *= 0.03 and 0.004, respectively). Low intakes of saturated, monounsaturated and polyunsaturated fatty acids were also associated with high proliferation (*P *= 0.02, 0.004 and 0.003, respectively). Low energy and unadjusted fat intakes were associated with cyclin D_1 _overexpression (*P *= 0.02 and 0.007, respectively), whereas cyclin E overexpression was positively correlated with fat intake. Oestrogen receptor status and expression of the tumour suppressor gene p27 were not associated with either diet or body constitution.

**Conclusion:**

Low energy and low total fat (polyunsaturated fatty acids in particular) intakes, and high body mass index were associated with relatively more malignant breast tumours. Dietary behaviours and body constitution may be associated with specific types of breast cancer defined by conventional pathology parameters and cyclin D_1 _and cyclin E expression. Further studies including healthy control individuals are needed to confirm our results.

## Introduction

The aetiology of breast cancer is complex, but a number of risk factors have been identified. In human studies body mass index (BMI) and hip and waist circumferences have been described as positively associated with risk for developing breast cancer [1]. Animal experiments support these findings and report that obesity enhances the development of mammary tumours [2]. To our knowledge, no studies have been conducted to evaluate whether there is an eventual association between obesity and tumour characteristics in animals.

The association between dietary intake and body composition is still under debate [3,4], and the biological link between diet and breast cancer is unclear. Dietary studies indicate that diet may contribute factors that either promote or protect against breast cancer [5]. High alcohol intake, for example, has been associated with breast cancer [1,6]. A large number of studies have examined the link between dietary fat and breast cancer, and most studies have found no or weak associations [7-9]. A recent attempt to clarify the hypothesis that a low-fat dietary pattern can reduce breast risk was made by Prentice and coworkers [10] in a randomized trial, but the results showed that risk for breast cancer did not differ between the two groups studied. The lack of any clear associations between diet and breast cancer in epidemiological studies could result from difficulties in dietary assessment, but they may also result from the fact that breast cancer is a heterogeneous disease that may have disparate genetic associations and different aetiological bases.

Breast cancer is heterogeneous in terms of pathology parameters such as tumour type, size, grade, hormone receptor status and lymph node involvement. These subgroups are related to clinical behaviours and prognoses [11]. Tumour proliferation, as reflected by the Ki67 index, is closely associated with tumour aggressiveness, and the Ki67 index is often considered an independent prognostic marker for breast cancer [12]. Another approach to defining subgroups of breast cancer is to map different gene aberrations in breast cancer. The tumour suppressor gene p27 acts as a cyclin-dependent kinase inhibitor [13]. Other gene aberrations related to defects in cell cycle control such as overexpression of cyclin D_1 _or cyclin E affect the cell cycle at the G_1_/S checkpoint and may affect chromosomal stability [14,15]. Indeed, these aberrations are common in cancer [16] and have even been proposed to be mandatory in tumour transformation processes [17]. In experimental animal studies [18] energy restriction was found to reduce mammary tumour cell proliferation via G_1 _cell cycle arrest, establishing a connection between dietary intake and cell cycle regulators.

The association between fat intake and breast cancer incidence overall was studied in previous work as part of the Malmö Diet and Cancer Study (MDCS), although with a shorter follow-up period and fewer breast cancer cases [19]. The aim of the present study was to explore the association between different subgroups of breast cancer and several dietary factors and body constitution. Incident breast cancer cases from the MDCS were classified according to tumour type, grade, oestrogen receptor (ER) status, expression of p27, cyclin D_1 _and cyclin E, and proliferation (Ki67).

## Materials and methods

### Study population

The MDCS is a population-based prospective cohort study [20]. Recruitment of the cohort was previously described in detail [21]. Individuals joined the study either spontaneously or after receiving an invitation by mail. Insufficient Swedish language skills was the only exclusion criterion. A total of 17,035 women born between 1923 and 1950 took part in the study and were examined during the period between 1991 and 1996.

Breast cancer cases were ascertained by record linkage with the Swedish Cancer Registry, and by the end of the follow-up cut-off date (31 December 2001) a total of 440 women had been diagnosed with incident breast cancer. Ethics permission for the MDCS was obtained from the Ethical Committee at Lund University (LU 51-90).

### Dietary data

Diet history data were obtained by combining a 7-day menu book ('current' diet information, concerning cooked meals and cold beverages, as recorded by the participants at home) and a diet history questionnaire ('usual' diet information, concerning foods consumed regularly but not eaten during cooked meals) in which the reference period was the preceding year. Dietary interviewers conducted a 1-hour interview focusing on food preparation and portion sizes, which were reported in the 7-day menu book, and checked the correctness of questionnaires that were completed at home. The reproducibility and concurrent validity were examined using 18 days of weighed food records collected over 1 year as the reference. Further details are described elsewhere [22,23]. The period during which the data were collected and by which dietary interviewer were recorded. A dichotomized variable based on the questionnaire item 'Have you substantially changed your dietary habits because of illness or any other reason?' was used to identify any recent change in dietary habit. The coding routines were altered halfway through the baseline examination period in order to reduce the interview time; this resulted in two method versions, referred to as the 'old' and 'new' versions [19]. The nutrient variables examined in this study were daily intake of total energy (J), total fat (g), protein (g), carbohydrate (g), saturated fatty acids (SFA; g), monounsaturated fatty acids (MUFA; g) and polyunsaturated fatty acids (PUFA; g).

### Baseline examination

During the baseline examinations all participants completed a dietary assessment and a lifestyle questionnaire, and had their anthropometric measurement factors recorded.

At baseline, hip and waist circumferences (m) were measured and BMI was calculated as kg/m^2^. Body composition was estimated using a single frequency bioimpedance method (BIA 103; RLJ Systems, Detroit, MI, USA) to measure body fat percentage. Alcohol habits were defined, based on the questionnaire, in four categories: abstainers, low consumers, medium consumers and high consumers. Smoking status was defined as current, former, or never. A physical activity score was obtained by multiplying the number of minutes used for each activity, from a list of 18 activities modified from of the Minnesota Leisure Time Physical Activity Instrument [24] with an activity-specific factor. Four categories of physical activity status were identified. These variables are described in detail elsewhere [25].

### Immunohistochemistry

Out of 440 women diagnosed with breast cancer, invasive tumour material was obtained from 346 cases. In 38 cases there was no available tissue. Fifty cases were diagnosed as ductal carcinoma *in situ *and therefore excluded. Six cases were biopsies only and not suitable for arraying. All cases were re-evaluated by one breast pathologist (LA) and tumour grading was performed according to the method proposed by Elston and Ellis [11]. For the construction of tissue microarays (TMAs), two 0.6 mm tissue cores were collected from each tumour block and arranged in a recipient block using a manual tissue arrayer (Beecher Inc., Sun Prairie, WI, USA). Slides were then processed in an automatic immunohistochemistry staining machine. The antibodies used were as follows: ER (pre-diluted anti-ER 6F11; Ventana, Tucson, AZ, USA), cyclin D_1 _(1:100 DCS-6; Dako, Glostrup, Denmark), cyclin E (1:100 HE12; Santa Cruz, CA, USA), p27 kip1 (1:200 SX53G8; Dako) and Ki67 (1:200 MIB-1; Dako). Tumours were grouped into no, low, medium, or high expression of ER, cyclin D_1_, cyclin E, p27 and Ki67, using the following respective cut-offs: 0% to 1%, 2% to 10%, 11% to 50% and 51% to 100% positive nuclei. To dichotomize the material, high and low categories were created for cyclin D_1 _and cyclin E (cut-off at 50%) and Ki67 (cut-off at 10%). All arrays were evaluated independently twice (SB), and in case of discrepancy a third examination was performed followed by a final decision.

### Statistical methods

All calculations were performed using SPSS version 11 (SPSS Institute, Chicago, IL, USA). Ln transformation of continuous variables (age, dietary intake and anthropometric factors) was carried out to normalize the data distribution. All statistical tests were two-sided and a *P *value less than 0.05 was considered statistically significant. Correlation analysis examined associations between pathology parameters. Analysis of variance was used to compare means of dietary and body measurements between different subgroups of breast tumours. All analyses were adjusted for age at baseline. Analyses concerning dietary intake were adjusted for diet assistant, the period of data collection, diet methodology (old or new method version) and recent food habit changes (yes or no), as well as for total energy intake when stated. It has been suggested that anthropometric factors may be associated in different directions with premenopausal and postmenopausal breast cancer. Hence, we repeated all the analyses of anthropometric factors separately for premenopausal and postmenopausal women.

## Results

The distribution of breast tumours revealed a predominance of ductal carcinomas (69%) as well as low-grade tumours (75% grade 1 and 2). In the TMAs, adequate tumour material was obtained in the majority of cases (91%), with a similar targeting rate for different tumour types (data not shown).

The distribution of demographic and breast cancer risk factors in the study population is illustrated in Table 1. Correlations between tumour type and grade as well as immunohistochemical expression of ER, cyclin D_1_, cyclin E, p27 and Ki67 are summarized in Table 2.

### Anthropometric measurements in relation to subgroups of breast cancer

Small hip circumference, low BMI and tall stature before breast cancer diagnosis were statistically significantly related to low-grade tumours (Table 3). Height was negatively associated with overexpression of cyclin E. In the separate analyses of premenopausal and postmenopausal women, a similar trend was seen in both groups, although with a slightly elevated *P *value for postmenopausal women and a nonsignificant result in premenopausal women (data not shown). There were no significant associations between objective body measurements and ER, cyclin D_1_, p27 and Ki67 when analysing the entire cohort. The separate analyses of premenopausal breast cancer exhibited a statistically significant association between high stature and tumours high in Ki67 and low in p27 (data not shown). Body fat percentage was not significantly associated with any specific subgroup of breast cancer.

### Energy and macronutrients intake in relation to sub-groups of breast cancer

Low energy intake was associated with nucleus grade 3 tumours as well as overexpression of cyclin D_1 _and high proliferation (Table 4). Low fat intake was associated with high proliferation and cyclin D_1 _overexpression, whereas high fat intake was associated with overexpression of cyclin E. Following adjustment for energy intake, the differences between subgroups were similar although less pronounced (Table 5). Intake of carbohydrates or protein was not associated with any subgroup of breast cancer.

Neither ER status nor the expression of the tumour suppressor gene p27 was associated with any of the dietary variables.

### Dietary fatty acids intake in relation to subgroups of breast cancer

Low intake of PUFA was associated with high proliferation (Tables 4 and 5), and in energy-adjusted analyses the result remained significant (data not shown; *P *= 0.04). Low intake of MUFA was associated with overexpression of cyclin D_1 _and high proliferation. Energy adjusted MUFA and proliferation were associated, albeit with borderline significance. Low intake of SFA was relatively more common in tumours of nucleus grade 3 as well as in tumours of high proliferation and with overexpression of cyclin D_1_, whereas overexpression of cyclin E was associated with high SFA intake. The relation to cyclin D_1 _remained significant with energy-adjusted SFA.

Smoking status, alcohol habits and physical activity were not associated with any specific subgroup of breast cancer (data not shown).

## Discussion

We found that low intakes of energy and total fat (especially PUFA), and high BMI were associated with more malignant breast cancer.

Some methodological issues must be addressed. Dietary assessment may be biased because of measurement error, for instance over- and under-reporting. The validity of dietary data in the MDCS has been examined using 18 days of weighed food records collected over 1 year in a subgroup of MDCS participants (126 men and 115 women) and validity was found to be high [22,23].

Whether energy-adjusted variables should be used when analyzing any relation between dietary intake and disease is a matter of controversy among nutritional researchers [26,27], and in this study we present results using the total, as well as energy-adjusted, intake of macronutrients.

A prevalent or subclinical breast cancer may affect anthropometric measurements and dietary habits. All data concerning objective body measurements were gathered between 0.2 and 10.4 (mean 4.3) years before breast cancer diagnosis, and the values were therefore most likely unaffected by disease. Supporting this interpretation is the lack of relation between BMI and time to diagnosis (*P *= 0.526, *r *= -0.021 [Spearman's correlation test]). Similar results were obtained for energy intake and time to diagnosis (*P *= 0.208, *r *= -0.060).

Tumour classification with regard to type and grade was performed according to current classification systems. The tissue microarray (TMA) technique used in this study is now a well documented method for high-throughput tissue screening, with two tissue cores considered a sufficient sampling amount [28,29]. The distribution of the immunohistochemical markers was in accordance with earlier studies [16], thus validating the assessments.

The participation rate in the MDCS was about 40% of the potential participant population. The participants did have a higher incidence of breast cancer compared with the source population [21] and were most likely a selected group in terms of socioeconomic factors. Nevertheless, the distribution of histological type and grade within the incident breast tumours in this study was similar to that in other studies [11,30]. Even if our breast cancer population were different from the background population, it would still be possible to make internal comparisons between different tumour groups in terms of dietary and anthropometric measurements.

Because several methodological factors in the MDCS may affect dietary measurements, all analyses were adjusted for diet assistant, period of data collection, diet methodology and past food habit change, as well as for total energy intake when stated. Hence, these factors ought not to have confounded dietary assessments. It was decided not to adjust the present analysis for established risk factors for breast cancer or factors that affect true dietary intake (for example socioeconomic index), because the main objective was to conduct perform a descriptive and exploratory analysis of dietary intake in different groups of breast cancer as defined by pathological and biological properties.

The present study includes a large number of comparisons, and *P *values should be interpreted with caution. Because no previous study has addressed the same issue using our methodology, we consider the present analyses as a first, hypothesis-generating study. Our findings need confirmation in future studies including healthy control individuals.

In this report we demonstrate an association between highly proliferative tumours and low energy intake, as well as low intakes of fat and PUFA. Several other human studies have reported that high energy intake is related to increased risk for breast cancer [31,32], but it is still unclear whether fat has a specific impact on breast cancer risk [7,9]. To our knowledge, no studies have reported on any association between diet and proliferation in breast cancer.

Furthermore, we found an association between low energy intake, particularly low fat intake, and overexpression of cyclin D_1_. Cyclin E, however, was positively associated with fat intake, but this association did not remain significant in the dichotomized analyses. The opposite behaviours for cyclin D_1 _and cyclin E are in accordance with earlier observations showing that cyclin E overexpressing tumours are low in cyclin D_1 _and *vice versa *[33]. Experimental animal studies support our findings of a connection between dietary intake and cell cycle regulators because energy restriction has been demonstrated to reduce mammary tumour cell proliferation via G_1 _cell cycle arrest, possibly through increased expression of p27 and reduced expression of cyclin D_1 _[18,34]. This may appear to be in conflict with our results, in which low energy intake was associated with cyclin D_1 _overexpression, and p27 did not exhibit any connection to energy intake. However, animal models using chemically induced breast tumours cannot readily be translated into a far more complex tumour genesis in humans, which presumably are influenced by a multitude of environmental factors.

Subgroups defined by ER were not linked to dietary behaviour or body composition. Some studies indicate that high fat diets are associated with increased risk for ER-positive tumours [35-37], whereas Verreault and coworkers [38] found no association between dietary fat and ER status. A few studies suggest that different types of PUFA may have opposing influences on breast cancer risk [39,40]. Studies concerning body constitution report an increased risk for ER-positive tumours in obese postmenopausal women who are supplied with oestrogens via conversion of androstendione to oesterone in fat tissues, which is in contrast to premenopausal women, whose primarily oestrogen source is the ovaries [41]. However, the present analyses include both premenopausal and postmenopausal women because there are only a limited number of cases in each subgroup, making further stratification into premenopausal and postmenopausal women unsuitable. This might explain the different results. Furthermore, analyses in the present study did not include healthy control individuals, which might also have contributed to the different results. However, in order to map potential differences in anthropometric risk factors in premenopausal and postmenopausal breast cancer, we performed separate analyses, which confirmed our initial results in the entire cohort. In addition, premenopausal breast tumours were characterized by high proliferation and low expression of p27 in tall women. The suppressor gene product p27 was not associated with any examined variable in the entire cohort. Few comparable human studies have addressed the relationship between p27 and dietary habits or body constitution; Daling and coworkers [42] examined the relationship between BMI and p27 but found no significant associations.

In contrast to fat, protein and carbohydrate were not significantly associated with any specific tumour characteristics. A recently published report [43] indicates an association between increased intake of carbohydrates and breast cancers with more favourable prognosis, which is consistent with the nonsignificant tendency observed in the present study.

The tumour-promoting effects of fat may be dependent on amount as well as type [44]. In the present study, the inverse relation between energy-adjusted PUFA and proliferation was most pronounced, indicating that low intake of PUFA corresponds with the occurrence of highly proliferative breast cancer. A previous study from the MDCS including 237 incident breast cancer cases found a positive association between high intake of PUFA, specifically ω 6 fatty acids, and breast cancer incidence [19]. Other studies analyzing the risk for breast cancer have indicated similar positive associations between PUFA and breast cancer incidence [19,45]. Human studies on the association between fatty acids and breast tumour characteristics have, to our knowledge, not yet been published, and further studies are needed in order to validate our preliminary findings.

Body composition, expressed in terms of BMI, waist and hip circumference, and height, was associated with tumour grade, and slender body constitution corresponded with low-grade tumours. The association between body constitution and breast cancer stage has been reported in other studies [46,47], and an association between obesity and high-grade tumours has also been described [42]. These observations support our findings.

In the present study body size and distribution of body fat were clearly associated with histological grade, whereas energy intake – with an emphasis on fat – appeared to influence the proliferation rate and overexpression of cell cycle regulators. The image of a slender figure with high energy and fat intake and a correspondence with a less malignant phenotype may seem controversial. However, similar to other epidemiological studies [3,48], energy and fat intake and body constitution were shown not to be significantly associated (data not shown). Notably, the variable 'total energy' should not be confused with 'energy balance', which takes energy expenditure into account [49]. This might explain why, in this study, high energy intake and a slender figure are both related to less aggressive tumours. Physical activity was nevertheless not associated with any specific subgroup of breast cancer, which could be caused by difficulties in assessing this variable [50]. Obese individuals commonly report low-energy diets [51] as well as recent changes in dietary habits [52], making it plausible that obese participants are in reality low in energy intake.

Alcohol intake was not associated with any of the breast cancer subgroups examined, but a previous report from the MDCS identified an association between high intake of wine or total alcohol (nonsignificant) and increased breast cancer risk [6].

## Conclusion

The above findings indicate that the strategy of subgrouping breast cancer according to proliferation, tumour grade and expression of cyclin D_1 _and E might be a useful approach when studying breast cancer in relation to dietary behaviours and body constitution. Further studies are needed to improve the delineation of women at risk for developing highly malignant breast cancer and, hopefully, to contribute to novel prevention strategies.

## Abbreviations

BMI = body mass index; ER = oestrogen receptor; MDCS = Malmö Diet and Cancer Study; MUFA = monounsaturated fatty acids; PUFA = polyunsaturated fatty acids; SFA = saturated fatty acids; TMA = tissue microarray.

## Competing interests

The authors declare that they have no competing interests.

## Authors' contributions

SB carried out the immunohistochemical assessment, performed the statistical analysis and drafted the manuscript. EW participated in the design of the study and interpretation of data, and helped to draft and revise the manuscript. KJ supervised the immunoassays and helped to draft the manuscript. LA re-evaluated tumours according to type and grade, and participated in the design of the study. BG conducted the statistical design and provided statistical support. GB participated in the design of the study. JM revised the manuscript critically. GL participated in the design of the study and interpretation of data, and helped to draft the manuscript. All authors read and approved the final manuscript.

## Figures and Tables

**Table 1:** Description of the study population

Factor (number of individuals for whom information was available)	Incident breast cancer (*n *= 440)
Age at baseline (years; 440)	57.2 (7.1)
Education (439)	70
O-level college	6
A-level college university	24
Type of occupation (431)	
Manual worker	34
Nonmanual worker	59
Employer/self-employed	7
Married/cohabiting (440)	
No	34
Yes	66
Age at menarche (years; 440)	
≤12	22
>12 to <15	52
≥15	26
Parity (number of children; 426)	
0	13
1	20
2	44
3	18
≥4	5
Age at first childbirth (years; 440)	
Nullipara	13
≤20	17
>20 to ≤25	34
>25 to ≤30	26
>30	10
Menopausal status (440)	
Premenopausal	25
Perimenopausal	7
Postmenopausal	68
Exposure to OC (439)	
Never	49
Ever	51
Current use of HRT (440)	
No	71
Yes	29
Body mass index (kg/m^2^; 440)	25.5 (4.1)
Alcohol consumption (440)	
Nothing last year (teetotaller)	11
Something last year (not last month)	11
Something last month	78
Smoking (440)	
Never	40
Current	30
Ex	30

Values are expressed as percentage or as mean (standard deviation). HRT, hormone replacement therapy; OC, oral contraceptives.

**Table 2:** Associations between tumour type, grade, ER, cyclin D_1_, cyclin E, p27 and proliferation (Ki67)

	Tumour grading	Nucleus grade	ER	Cyclin D_1_	Cyclin E	p27	Ki67
Tumour type^a^	(<0.001**)	(<0.001**)	(0.01*)	(0.11)	(0.10)	(0.67)	(0.009**)
Tumour grading	1 (-)	0.789 (<0.001**)	-0.374 (<0.001**)	0.082 (0.155)	0.339 (<0.001**)	-0.271 (<0.001**)	0.530 (<0.001**)
Nucleus grade		1 (-)	-0.369 (<0.001**	0.050 (0.391)	0.323 (<0.001**)	-0.188 (0.001)	0.448 (<0.001**)
ER			1 (-)	0.154 (0.007**)	-0.540 (<0.001**)	0.291 (<0.001**)	-0.282 (<0.001**)
Cyclin D_1_				1 (-)	0.024 (0.674)	0.262 (<0.001**)	0.288 (<0.001**)
Cyclin E					1 (-)	-0.094 (0.099)	0.395 (<0.001**)
p27						1 (-)	-0.211 (<0.001**)
Ki67							1 (-)

*P *values are given in parentheses. ^a^For tumour type (ductal, lobular, tubular), the χ^2 ^test was used. Otherwise, Pearson's correlation test was used. *P < 0.05, **P < 0.01. ER, oestrogen receptor.

**Table 3:** Mean values for anthropometric measurements in specific subgroups of breast cancer

	*n*	Height (m)	BMI (kg/m^2^)	Hip (m)	Waist (m)	Body fat (%)
PAD						
*P *value		0.98	0.69	0.19	0.16	0.82
Ductal	239	1.64	25	0.98	0.78	31
Lobular	72	1.64	25	0.98	0.77	30
Tubular	22	1.64	25	0.95	0.75	31
Others^a^	13	-	-	-	-	-
Grade						
*P *value		0.01*	0.03*	0.009**	0.18	0.06
Grade 1	95	1.66	24	0.96	0.76	30
Grade 2	164	1.64	26	0.99	0.79	31
Grade 3	85	1.64	25	0.98	0.78	31
Nucleus grade						
*P *value		0.08	0.11	0.02*	0.27	0.19
Nuclear grade 1	52	1.65	24	0.95	0.76	30
Nuclear grade 2	176	1.63	25	0.99	0.78	30
Nuclear grade 3	115	1.63	26	0.98	0.78	31
ER						
*P *value		0.62	0.33	0.14	0.41	0.39
0–1%	27	1.64	25	0.96	0.76	30
2–10%	4	1.63	27	1.02	0.78	32
11–50%	12	1.62	27	1.03	0.82	33
51–100%	277	1.64	25	0.98	0.78	30
Cyclin D_1_						
*P *value		0.84	0.16	0.13	0.35	0.46
0–1%	208	1.64	25	0.98	0.78	30
2–10%	64	1.64	26	0.99	0.79	31
11–50%	27	1.64	25	0.97	0.76	31
51–100%	12	1.63	24	0.93	0.74	30
Cyclin E						
*P *value		0.002**	0.75	0.81	0.82	0.97
0–1%	251	165	25	0.98	0.78	30
2–10%	40	162	25	0.98	0.77	31
11–50%	16	161	26	0.98	0.79	31
51–100%	10	163	25	0.95	0.75	31
p27						
*P *value		0.19	0.39	0.16	0.10	0.08
0–1%	90	165	25	0.98	0.78	31
2–10%	50	164	26	1.00	0.80	32
11–50%	71	163	25	0.96	0.76	30
51–100%	102	164	25	0.97	0.77	30
Ki67						
*P *value		0.90	0.31	0.17	0.59	0.10
0–1%	130	164	25	0.97	0.77	30
2–10%	93	164	26	0.99	0.79	31
11–50%	57	165	25	0.97	0.77	30
51–100%	33	164	26	0.97	0.77	31

^a^Mucinous, medullary, and intracystic breast tumours were not included in statistical analyses because of their small number. *P < 0.05; **P < 0.01. ER, oestrogen receptor; PAD, pathological anatomical diagnosis.

**Table 4:** Means of intake of energy, macronutrients, and fatty acids in relation to specific subgroups of breast cancer

	Energy (Joule/day)	Total fat (g/day)	Total carbohydrates (g/day)	Total protein (g/day)	Total alcohol (g/day)	SFA (g/day)	MUFA (g/day)	PUFA (g/day)
PAD								
*P *value	0.82	0.56	0.84	0.94	0.12	0.86	0.34	0.27
Ductal	1955	82	212	76	1.0	35	29	13
Lobular	1929	79	210	75	1.9	34	27	12
Tubular	2006	82	219	77	2.6	34	28	13
Others^a^								
Grade								
*P *value	0.09	0.20	0.39	0.02*	0.28	0.16	0.34	0.40
1	2002	83	216	78	1.7	35	29	13
2	1966	82	212	77	1.3	35	29	13
3	1852	77	204	71	0.9	32	27	12
Nucleus grade								
*P *value	0.02*	0.07	0.06	0.02*	0.10	0.04*	0.16	0.48
1	2012	83	217	78	2.5	36	29	13
2	2002	84	217	78	1.1	36	29	13
3	1846	77	201	72	1.0	32	27	12
ER								
*P *value	0.90	0.75	0.77	0.80	0.80	0.73	0.55	0.98
0	1955	81	219	72	0.9	33	29	13
1	1794	74	188	76	3.7	31	25	12
2	1895	75	214	77	1.3	31	25	13
3	1954	82	211	76	1.3	35	29	13
Cyclin D_1_								
*P *value	0.11	0.06	0.37	0.12	0.56	0.02*	0.046*	0.47
0	1968	82	213	77	1.3	35	29	13
1	1943	82	208	74	1.4	34	29	13
2	1891	80	206	71	0.6	36	28	12
3	1651	64	185	67	0.6	25	22	11
Cyclin E								
*P *value	0.10	0.03*	0.43	0.25	0.40	0.04*	0.05	0.14
0	1963	82	212	76	1.4	35	29	13
1	1786	72	198	71	0.6	30	26	12
2	2016	83	222	79	1.6	34	30	13
3	2139	98	222	81	1.0	42	34	16
p27								
*P *value	0.49	0.93	0.12	0.43	0.78	0.98	0.96	0.69
0	1970	81	216	75	1.3	34	29	13
1	1947	81	217	75	0.8	34	28	13
2	1876	80	197	73	1.3	34	28	12
3	1988	83	214	78	1.3	35	29	13
Ki67								
*P *value	0.16	0.02*	0.78	0.40	0.27	0.02*	0.02*	0.02*
0	1970	83	210	77	1.6	35	29	13
1	2002	85	214	77	1.4	36	30	13
2	1835	72	206	72	0.7	30	25	12
3	1872	79	204	73	0.8	35	28	11

^a^Mucinous, medullary and intracystic breast tumours were not included in statistical analyses because of their small number. *P < 0.05; **P < 0.01. ER, oestrogen receptor; MUFA, monounsaturated fatty acids; PUFA, polyunsaturated fatty acids; PAD, pathological anatomical diagnosis; SFA, saturated fatty acids.

**Table 5:** Means of intake of energy, macronutrients, and fatty acids in relation to specific subgroups of breast cancer

	Energy (Joules/day)	Total fat (g/day)	Energy-adjusted fat (g/day)	SFA (g/day)	Energy-adjusted SFA (g/day)	MUFA (g/day)	Energy-adjusted MUFA (g/day)	PUFA (g/day)	Energy-adjusted PUFA (g/day)
Cyclin D_1_^a^									
*P *value	0.02*	0.007**	0.16	0.002*	0.04*	0.006**	0.13	0.32	0.31
Low	1957	82	81	35	34	29	28	13	13
High	1652	63	76	25	30	22	26	11	13
Cyclin E^b^									
*P *value	0.26	0.07	0.09	0.09	0.18	0.08	0.12	0.07	0.15
Low	1937	80	81	34	34	28	28	13	13
High	2123	97	88	42	38	34	31	15	14
Ki67^c^									
*P *value	0.029*	0.004**	0.048*	0.019*	0.315	0.004**	0.058	0.003**	0.053
Low	1984	84	82	35	35	29	29	13	13
High	1850	75	79	32	34	26	28	12	12

All analyses are adjusted for age, diet assistant, period, method version and changed dietary habits. Energy adjustment is applied where stated. ^a^Cyclin D_1 _overexpression with nucleus fraction ≥50%. ^b^Cyclin E overexpression with nucleus fraction ≥50%. ^c^High proliferation index with nuclear fraction ≥10. *P < 0.05; **P < 0.01. MUFA, monounsaturated fatty acids; PUFA, polyunsaturated fatty acids; SFA, saturated fatty acids.

## References

[B1] Key TJ, Schatzkin A, Willett WC, Allen NE, Spencer EA, Travis RC (2004). Diet, nutrition and the prevention of cancer. Public Health Nutr.

[B2] Hakkak R, Holley AW, Macleod SL, Simpson PM, Fuchs GJ, Jo CH, Kieber-Emmons T, Korourian S (2005). Obesity promotes 7,12-dimethylbenz(a)anthracene-induced mammary tumor development in female zucker rats. Breast Cancer Res.

[B3] Willett WC (2002). Dietary fat plays a major role in obesity: no. Obes Rev.

[B4] Astrup A, Ryan L, Grunwald GK, Storgaard M, Saris W, Melanson E, Hill JO (2000). The role of dietary fat in body fatness: evidence from a preliminary meta-analysis of ad libitum low-fat dietary intervention studies. Br J Nutr.

[B5] Fung TT, Hu FB, Holmes MD, Rosner BA, Hunter DJ, Colditz GA, Willett WC (2005). Dietary patterns and the risk of postmenopausal breast cancer. Int J Cancer.

[B6] Mattisson I, Wirfalt E, Wallstrom P, Gullberg B, Olsson H, Berglund G (2004). High fat and alcohol intakes are risk factors of postmenopausal breast cancer: a prospective study from the Malmo diet and cancer cohort. Int J Cancer.

[B7] Cho E, Spiegelman D, Hunter DJ, Chen WY, Stampfer MJ, Colditz GA, Willett WC (2003). Premenopausal fat intake and risk of breast cancer. J Natl Cancer Inst.

[B8] Hunter DJ, Spiegelman D, Adami HO, Beeson L, van den Brandt PA, Folsom AR, Fraser GE, Goldbohm RA, Graham S, Howe GR (1996). Cohort studies of fat intake and the risk of breast cancer: a pooled analysis. N Engl J Med.

[B9] Velie E, Kulldorff M, Schairer C, Block G, Albanes D, Schatzkin A (2000). Dietary fat, fat subtypes, and breast cancer in postmenopausal women: a prospective cohort study. J Natl Cancer Inst.

[B10] Prentice RL, Caan B, Chlebowski RT, Patterson R, Kuller LH, Ockene JK, Margolis KL, Limacher MC, Manson JE, Parker LM (2006). Low-fat dietary pattern and risk of invasive breast cancer: the Women's Health Initiative Randomized Controlled Dietary Modification Trial. JAMA.

[B11] Elston CW, Ellis IO (1991). Pathological prognostic factors in breast cancer. I. The value of histological grade in breast cancer: experience from a large study with long-term follow-up. Histopathology.

[B12] Bouzubar N, Walker KJ, Griffiths K, Ellis IO, Elston CW, Robertson JF, Blamey RW, Nicholson RI (1989). Ki67 immunostaining in primary breast cancer: pathological and clinical associations. Br J Cancer.

[B13] Chiarle R, Pagano M, Inghirami G (2001). The cyclin dependent kinase inhibitor p27 and its prognostic role in breast cancer. Breast Cancer Res.

[B14] Nelsen CJ, Kuriyama R, Hirsch B, Negron VC, Lingle WL, Goggin MM, Stanley MW, Albrecht JH (2005). Short term cyclin D1 overexpression induces centrosome amplification, mitotic spindle abnormalities, and aneuploidy. J Biol Chem.

[B15] Lingle WL, Barrett SL, Negron VC, D'Assoro AB, Boeneman K, Liu W, Whitehead CM, Reynolds C, Salisbury JL (2002). Centrosome amplification drives chromosomal instability in breast tumor development. Proc Natl Acad Sci USA.

[B16] Nielsen NH, Loden M, Cajander J, Emdin SO, Landberg G (1999). G1-S transition defects occur in most breast cancers and predict outcome. Breast Cancer Res Treat.

[B17] Hanahan D, Weinberg RA (2000). The hallmarks of cancer. Cell.

[B18] Zhu Z, Jiang W, Thompson HJ (1999). Effect of energy restriction on the expression of cyclin D1 and p27 during premalignant and malignant stages of chemically induced mammary carcinogenesis. Mol Carcinog.

[B19] Wirfalt E, Mattisson I, Gullberg B, Johansson U, Olsson H, Berglund G (2002). Postmenopausal breast cancer is associated with high intakes of omega6 fatty acids (Sweden). Cancer Causes Control.

[B20] Berglund G, Elmstahl S, Janzon L, Larsson SA (1993). The Malmo Diet and Cancer Study. Design and feasibility. J Intern Med.

[B21] Manjer J, Carlsson S, Elmstahl S, Gullberg B, Janzon L, Lindstrom M, Mattisson I, Berglund G (2001). The Malmo Diet and Cancer Study: representativity, cancer incidence and mortality in participants and non-participants. Eur J Cancer Prev.

[B22] Callmer E, Riboli E, Saracci R, Akesson B, Lindgarde F (1993). Dietary assessment methods evaluated in the Malmo food study. J Intern Med.

[B23] Riboli E, Elmstahl S, Saracci R, Gullberg B, Lindgarde F (1997). The Malmo Food Study: validity of two dietary assessment methods for measuring nutrient intake. Int J Epidemiol.

[B24] Taylor HL, Jacobs DR, Schucker B, Knudsen J, Leon AS, Debacker G (1978). A questionnaire for the assessment of leisure time physical activities. J Chronic Dis.

[B25] Wallstrom P, Wirfalt E, Janzon L, Mattisson I, Elmstahl S, Johansson U, Berglund G (2000). Fruit and vegetable consumption in relation to risk factors for cancer: a report from the Malmo Diet and Cancer Study. Public Health Nutr.

[B26] Willett WC, Howe GR, Kushi LH (1997). Adjustment for total energy intake in epidemiologic studies. Am J Clin Nutr.

[B27] Kipnis V, Midthune D, Freedman L, Bingham S, Day NE, Riboli E, Ferrari P, Carroll RJ (2002). Bias in dietary-report instruments and its implications for nutritional epidemiology. Public Health Nutr.

[B28] Camp RL, Charette LA, Rimm DL (2000). Validation of tissue microarray technology in breast carcinoma. Lab Invest.

[B29] Hoos A, Cordon-Cardo C (2001). Tissue microarray profiling of cancer specimens and cell lines: opportunities and limitations. Lab Invest.

[B30] Sundquist M, Thorstenson S, Brudin L, Nordenskjold B (1999). Applying the Nottingham Prognostic Index to a Swedish breast cancer population. South East Swedish Breast Cancer Study Group. Breast Cancer Res Treat.

[B31] Chang SC, Ziegler RG, Dunn B, Stolzenberg-Solomon R, Lacey JV, Huang WY, Schatzkin A, Reding D, Hoover RN, Hartge P, Leitzmann MF (2006). Association of energy intake and energy balance with postmenopausal breast cancer in the prostate, lung, colorectal, and ovarian cancer screening trial. Cancer Epidemiol Biomarkers Prev.

[B32] Malin A, Matthews CE, Shu XO, Cai H, Dai Q, Jin F, Gao YT, Zheng W (2005). Energy balance and breast cancer risk. Cancer Epidemiol Biomarkers Prev.

[B33] Nielsen NH, Emdin SO, Cajander J, Landberg G (1997). Deregulation of cyclin E and D1 in breast cancer is associated with inactivation of the retinoblastoma protein. Oncogene.

[B34] Thompson HJ, Zhu Z, Jiang W (2003). Dietary energy restriction in breast cancer prevention. J Mammary Gland Biol Neoplasia.

[B35] Jain M, Miller AB (1997). Tumor characteristics and survival of breast cancer patients in relation to premorbid diet and body size. Breast Cancer Res Treat.

[B36] Kushi LH, Potter JD, Bostick RM, Drinkard CR, Sellers TA, Gapstur SM, Cerhan JR, Folsom AR (1995). Dietary fat and risk of breast cancer according to hormone receptor status. Cancer Epidemiol Biomarkers Prev.

[B37] Harlan LC, Coates RJ, Block G, Greenberg RS, Ershow A, Forman M, Austin DF, Chen V, Heymsfield SB (1993). Estrogen receptor status and dietary intakes in breast cancer patients. Epidemiology.

[B38] Verreault R, Brisson J, Deschenes L, Naud F, Meyer F, Belanger L (1988). Dietary fat in relation to prognostic indicators in breast cancer. J Natl Cancer Inst.

[B39] Jakovljevic J, Touillaud MS, Bondy ML, Singletary SE, Pillow PC, Chang S (2002). Dietary intake of selected fatty acids, cholesterol and carotenoids and estrogen receptor status in premenopausal breast cancer patients. Breast Cancer Res Treat.

[B40] Hislop TG, Kan L, Coldman AJ, Band PR, Brauer G (1988). Influence of estrogen receptor status on dietary risk factors for breast cancer. CMAJ.

[B41] Enger SM, Ross RK, Paganini-Hill A, Carpenter CL, Bernstein L (2000). Body size, physical activity, and breast cancer hormone receptor status: results from two case-control studies. Cancer Epidemiol Biomarkers Prev.

[B42] Daling JR, Malone KE, Doody DR, Johnson LG, Gralow JR, Porter PL (2001). Relation of body mass index to tumor markers and survival among young women with invasive ductal breast carcinoma. Cancer.

[B43] Giles GG, Simpson JA, English DR, Hodge AM, Gertig DM, Macinnis RJ, Hopper JL (2006). Dietary carbohydrate, fibre, glycaemic index, glycaemic load and the risk of postmenopausal breast cancer. Int J Cancer.

[B44] Wynder EL, Cohen LA, Muscat JE, Winters B, Dwyer JT, Blackburn G (1997). Breast cancer: weighing the evidence for a promoting role of dietary fat. J Natl Cancer Inst.

[B45] Wolk A, Bergstrom R, Hunter D, Willett W, Ljung H, Holmberg L, Bergkvist L, Bruce A, Adami HO (1998). A prospective study of association of monounsaturated fat and other types of fat with risk of breast cancer. Arch Intern Med.

[B46] Cui Y, Whiteman MK, Flaws JA, Langenberg P, Tkaczuk KH, Bush TL (2002). Body mass and stage of breast cancer at diagnosis. Int J Cancer.

[B47] Kim YA, Lee CW (2004). Effects of obesity on breast cancer stage at diagnosis in Korean women. Eur J Cancer Prev.

[B48] Willett WC, Leibel RL (2002). Dietary fat is not a major determinant of body fat. Am J Med.

[B49] Willett WC (1998). Nutritional Epidemiology.

[B50] Mattisson I, Wirfalt E, Aronsson CA, Wallstrom P, Sonestedt E, Gullberg B, Berglund G (2005). Misreporting of energy: prevalence, characteristics of misreporters and influences on observed risk estimates in the Malmö Diet and Cancer cohort. Br J Nutr.

[B51] Wirfalt E, Mattisson I, Gullberg B, Berglund G (2000). Food patterns defined by cluster analysis and their utility as dietary exposure variables: a report from the Malmo Diet and Cancer Study. Public Health Nutr.

[B52] Sonestedt E, Wirfalt E, Gullberg B, Berglund G (2005). Past food habit change is related to obesity, lifestyle and socio-economic factors in the Malmo Diet and Cancer Cohort. Public Health Nutr.

